# The Effect of the Physical and Chemical Properties of Synthetic Fabrics on the Release of Microplastics during Washing and Drying

**DOI:** 10.3390/polym14163384

**Published:** 2022-08-18

**Authors:** Sola Choi, Juhea Kim, Miyeon Kwon

**Affiliations:** Material & Component Convergence R&D Department, Korea Institute of Industrial Technology, Ansan 15588, Korea

**Keywords:** synthetic fabrics, microplastics, environmental pollution, washing, drying

## Abstract

Synthetic fibers released during washing are the primary source of microplastic pollution. Hence, research on reducing the release of microplastic fibers during washing has recently attracted considerable attention. As a result of previous studies, there is a difference in the amount of microplastic emission according to various types of fabrics. To mitigate the release of microplastics, the study of the reason for the difference in the amount of microplastics is needed. Therefore, this study investigated different synthetic fabrics that release microplastics and the physical properties of the fabrics that affect the release of fibers. Three types of fabrics with different chemical compositions were analyzed. The washing and drying processes were improved by focusing on the mechanical factors that affected microplastic release. Furthermore, based on the mass of the collected microplastic fibers, it was found that the chemical compositions of the fabric can affect the microplastics released during washing and drying. This evaluation of physical properties helped to identify the physical factors that affect microplastic release. These results may provide a basis for reducing microplastic fiber types, thereby minimizing unintended environmental pollution.

## 1. Introduction

Plastic production has increased from 0.5 million tons in 1950 to 367 million tons in 2019 [1], leading to increasing plastic waste in marine and inland waters. Plastic materials introduced into the environment do not degrade due to wear and weather. In addition, wind effects, wave actions, and plastic density affect the spread of plastic debris [2]. The United Nations Environment Program estimates that up to 18,000 pieces of plastic debris float on every square kilometer of the ocean [3,4]. The debris, composed of synthetic polymers, is more serious than other types of debris because it can remain in the marine environment for years due to its durability and slow degradation rate [4,5].

Some of the fragments in the debris are microplastics, which are small or fine (length < 5 mm) solid particles made of synthetic polymers. Fiber-shaped microplastics range from 3 to 15 mm in length and have a length-to-diameter ratio greater than 3 [6]. Microplastics have been reported on 18 shores across 6 continents, with a tendency for fibrous shapes and maximum concentrations of 124 fibers L^−1^. When Daphnia magna was exposed to polyethylene terephthalate microfibers (length: 62–1400 μm) at a concentration of 12.5–100 mg/L for 48 h, lethality increased due to ingestion [7]. Therefore, it is necessary to change the perception of microplastic collection, reduce the production of microplastics that pose a risk to the environment and human health, analyze the microplastics generated from fibers, and develop collection methods.

The direct evaluation of washing effluents as a source of microplastic fibers and the quantification of their release during washing have been studied. In addition, differences in the amounts of microplastics released according to various physicochemical washing parameters, such as the washing machine type, detergent used, and temperature, have been compared. The washing machine type was compared using actual domestic washing machines (top- and front-load washing machines) and an experimental washing machine (Launder-O-meter) [8,9,10]. Launder-O-meter produces approximately 40 times more microplastics than domestic washing machines [11]. In addition, previous studies indicate that the amounts of generated microplastics increased when detergents were utilized [4,9,12,13]. Moreover, little difference was found in the amount of discharged microplastics at temperatures below 44 °C [4,9,11,14,15]. Previous studies on the release of microplastics during washing have considered various fabric factors, such as the fabric construction (woven, knit, or nonwoven), yarn type (twisting, evenness, hairiness, and number of fibers), and processing history (spinning, knitting, weaving, scouring, bleaching, dyeing, finishing, and drying processes) of fibers [4,8,12,16].

In many studies, the generation of microplastics has differed depending on the fabric type. To mitigate microplastic release, it is necessary to understand the difference between the amounts of microplastics released from various fabrics. Fabric movements during washing and drying are caused by fabric migration, friction, shape transformation, and restoration [17]. That is, the movement of fabric can be affected by its physical properties, such as tensile and shear properties, bending, compression, surface properties, stiffness, weight, and thickness [17]. Thus, it can be inferred that when subjected to an external force during washing and drying, the difference between the amount of the released microplastics depends on the physical properties of the fabric. Therefore, in this study, the physical properties of fabrics were evaluated to analyze the relationship between the amount of microplastics and the fabric type.

Microplastic fibers released during synthetic fabric washing are the primary source of microplastic pollution. Previous studies have revealed the differences in the amount of the released microplastics based on various fabrics. To mitigate the release of microplastics, it is necessary to understand the reason for these differences. Hence, this study investigated synthetic fabrics with different chemical compositions to identify the physical properties of the fabrics that affect fiber release. Our study aimed to (1) observe the variation in the amount of the released microplastics during each washing and drying procedure, (2) quantify the microfibers released from fabrics with different chemical compositions by controlling other textile parameters, including the fabric construction and the yarn type, and (3) determine the physical properties of a fabric that affect microplastic generation. Furthermore, drying parameters that affect the release of microplastics were investigated. Focusing the study on fabric properties by strictly controlling other textile parameters will facilitate a determination of the factors that ultimately affect fiber release.

## 2. Materials and Methods

### 2.1. Materials

**Figure 1:**
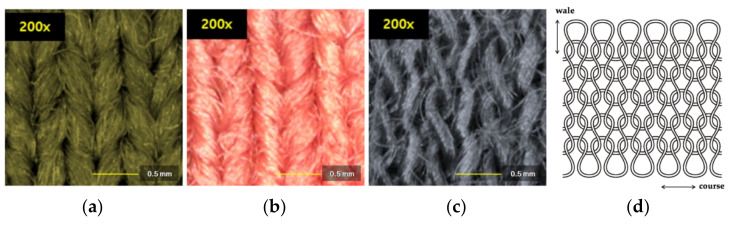
Images of the three fabric specimens. Front of single jersey fabrics consisting of (**a**) polyester knit, (**b**) nylon knit, and (**c**) acrylic knit. (**d**) The fabric construction of single jersey knit.

**Figure 2:**
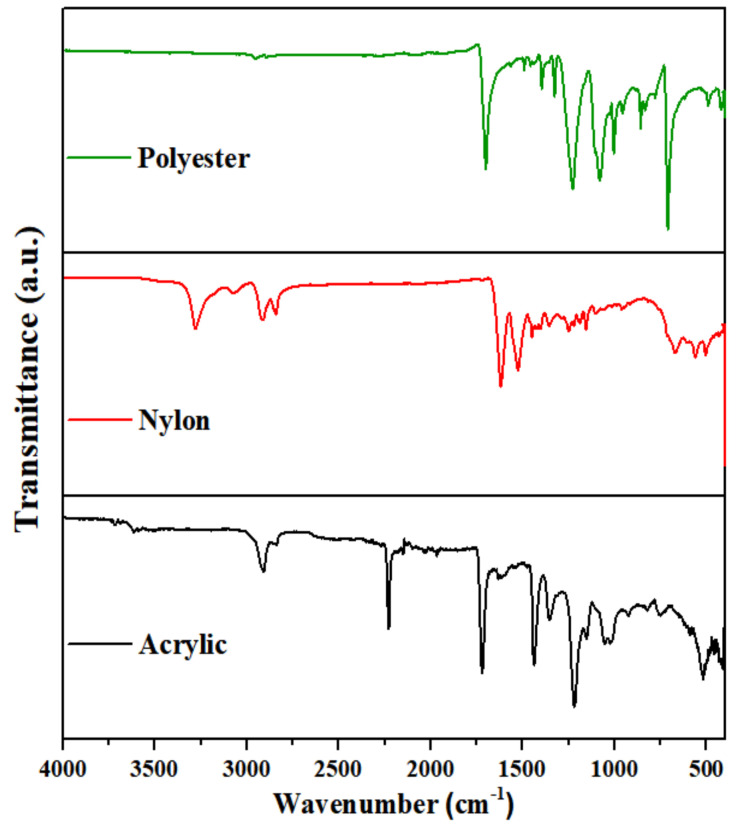
FTIR spectra of the three fabric specimens.

Three samples with different chemical compositions and different colors but similar fabric constructions were selected: polyester (green), nylon (red), and acrylic (black). The polyester fabric was purchased from Hwan Tex (Seoul, Korea), the nylon fabric from T World (Seoul, Korea), and the acrylic fabric from The One Tex (Seoul, Korea). The fabric construction types were spun yarn and single jersey knit, which have a basic knit construction (Figure 1). Table 1 shows the fabric characteristics obtained by measuring the specimen thickness and weight according to ISO 4603 and ISO 3801, respectively. As commercially available fabrics were selected as specimens, a compositional analysis was performed to accurately identify the components of the three types of synthetic fibers (Figure 2). Each fabric type was identified using Fourier transform infrared spectroscopy (FT-IR) (Vertex 80v, Bruker, Billerica, MA, USA). The obtained spectra were compared to a spectral database of synthetic polymers. FT-IR measured the spectral range from 500 cm^−1^ to 4000 cm^−1^ for sample spectral collection, and the FT-IR used was the attuned total reflection (ATR) technique. Unlike previous studies in which samples of the same size were used, samples of the same weight were utilized in this study [18,19]. Therefore, 500 g of fabric (corresponding to fiber pieces of fabrics, each weighing 100 g) was washed three times during each cycle. The edges of each specimen were sewn with white yarn to prevent excess loss of fibers.

### 2.2. Physical Properties

For each specimen, three physical properties were tested: fuzziness and pilling, tensile strength and elongation, and stiffness. These are the typical physical properties of fabrics that could affect the release of microplastics. The surface propensity to fuzziness and pilling was tested according to the ISO 12945-2:2000 method, also known as the Martindale method. The tested specimen generated artificial friction on the fabric surface, which was evaluated by assigning grades according to a visual evaluation of the surface change. The grading was based on the fuzziness and pilling visual evaluation specified in the ISO 12945-2:2000 standard. The grades can range from 1 to 5, with grade 5 being assigned when no surface change is detected after the test. The stiffness of the fabric is its resistance to deformation against the movement in the washing machine, and the amount of microplastics released is affected by the flexural stiffness of the fabric. To evaluate the stiffness of the fabric, a cantilever test was performed according to the ISO 4604 test method. The fabric was cut to obtain four specimens, each with a size of 2.5 × 15 cm in the wale and course directions. The flexural stiffness was calculated as follows. The test equipment had an inclination of 41.5°. The specimen was allowed to bend along the inclined surface. When the leading edge of the specimen touched the inclined surface, the length of the specimen from the horizontal surface of the equipment to the top edge of the specimen was measured. This value was divided by the weight of the specimen to obtain the flexural stiffness. The tensile strength and elongation of the fabric were tested according to the ISO 13934-2 test method, and the physical properties of the fabric in the wale and course directions were measured. The tensile strength is the force applied to the fabric per unit area, and the elongation is the rate of change in the length of the fabric due to strength.

### 2.3. Washing and Drying

**Figure 3:**
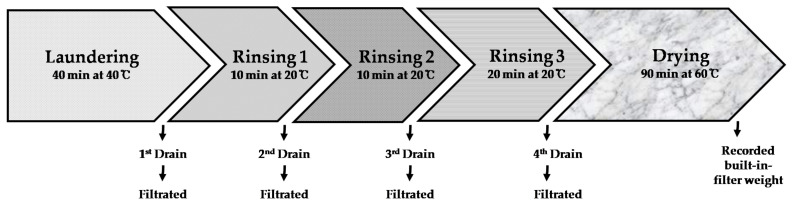
Washing procedures during one standard washing course and drying procedure.

Washing was carried out in a front-load washing machine (F9WK, LG Electronics, Seoul, Republic of Korea) using a standard washing course. A standard washing course is considered to have one laundering procedure and three rinsing procedures, and wastewater from each washing procedure was filtered to confirm the difference in the microplastic release as washing proceeded (Figure 3). The temperature of the laundering and rinsing processes were 40 ± 2 °C and 20 ± 2 °C, respectively. Washing was performed for a total of 1 h and 20 min, with 40 min for laundering, 10 min for rinsing processes 1 and 2, and 20 min for rinsing process 2. The fabrics were washed with tap water (pH 7), and detergent was not used. After completion of the experiment, the washing machine was emptied three times to eliminate microplastic residue or other contaminants. After washing, the fabrics were dried using a dual inverter heat-pump-type drum dryer (RH9WGN, LG Electronics, Seoul, Korea) with a standard drying course for 100 min at 60 °C. After the experiment, the empty machines were washed and dried three times to eliminate residual microplastics or other contaminants.

### 2.4. Filtration and Analysis

Quantitative filter papers (Grade 30, Hyundai Micro, Seoul, Korea) with 5 µm pore size, which is smaller than the diameter of microfibers, and a diameter of 185 mm were used. The wastewater, collected in 20 L containers for each washing procedure, was filtered separately. A specific filtration method reported by Choi et al. [20] was used. The weight and length of the microplastics were also analyzed. Gravimetric analysis was performed, and the parameters of the microplastics were calculated using the parts per million (mg/kg) equations, which represent the mass of the collected microplastics per unit mass of the textile. The weight of the microplastics was measured using a precision balance after drying on filter paper at a temperature of 26 ± 2 °C and a relative humidity of 20% for 24 h. The mass of collected materials per textile mass *ppm* was calculated using Equation (1):

*ppm* (mg/kg) = (*M*_m_ × 1000)/*M*_kg_ (1)

where *ppm* is the mass of the collected microplastics per textile mass (mg/kg), *M*m is the mass of microplastics collected during washing and drying (mg), and *M*kg is the mass of the test specimen (kg). The amount of microplastics released during drying was measured using the weight of the built-in filter before and after drying. Images of the filter taken by an image analyzer (magnification of 40×) were used to identify the fibers. The Image J program (National Institutes of Health, Bethesda, MD, USA) was used to analyze the fiber length. In the washing experiments, the length of the microplastics contained in the filter paper from each of the three samples was measured using a digital microscope (magnification of 40×). The captured fibers were spread evenly on the filter paper. The fiber lengths and diameters were analyzed using the Image J program.

### 2.5. Statistics

The SPSS Statistics program was used for statistical analysis. One-way analysis of variance (ANOVA) was carried out to determine whether differences existed in the amount of microplastics generated by each fabric composition, and the differences between groups were post-hoc tested using the Scheffe test. To determine the relative influence of the release of microplastics on pilling, stiffness, and tensile strength, Pearson’s correlation analysis was carried out to determine the correlation between two variables.

## 3. Results and Discussion

### 3.1. Analysis of Microplastics

**Figure 4:**
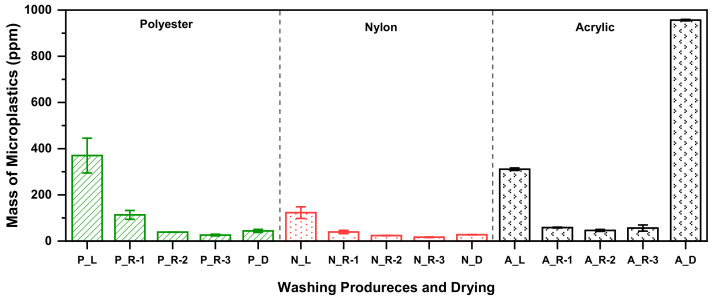
Mass of microplastics obtained during washing and drying (P, N, and A correspond to polyester, nylon, and acrylic fabrics, respectively; L is the laundering process; R-1, R-2, and R-3 are the rinsing processes; and D is the drying process).

**Figure 5:**
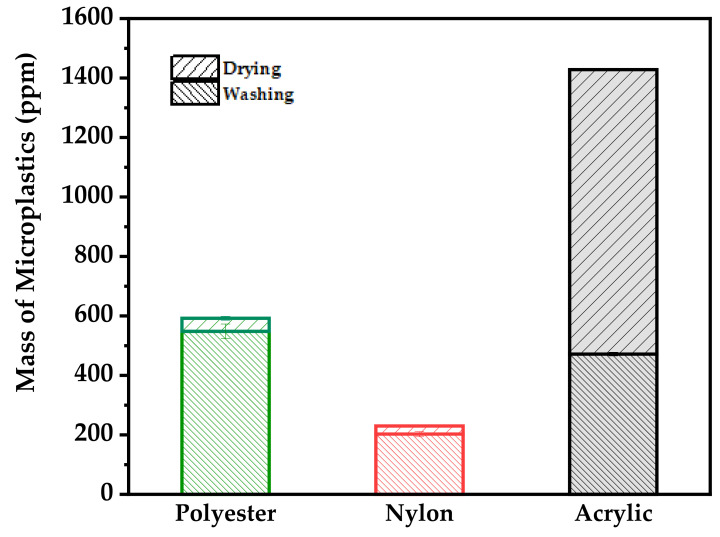
The total mass of microplastics generated by washing and drying.

The amounts of microplastic released from three different fabrics during washing and drying procedures were quantified. The release process of microplastics during washing and drying is shown in Figure 4. The release of microplastics by each fabric during washing and drying was quantified by weight (Figure 5). 

All the results showed a steady decrease in the overall loss of fibers during the washing procedures. However, for the acrylic fabric, the amount of microplastics generated by drying was 50% larger than that generated by washing.

The decrease in microplastic generation as washing proceeded is attributed to the influence of the washing time and temperature. It can be inferred that most microplastics were removed during the initial laundering procedure. Thus, their generation gradually decreased. This result was consistent with the sequential washing results obtained in other studies [21]. Sequential washing has been shown to consistently decrease the amount of microplastic fibers released as the number of washing cycles increases until the released amount reaches a constant level. More microplastics were released during laundering than during rinsing. This is because the time and temperature for laundering (40 min at 40 °C) were greater than those for the rinsing procedures (10 min for rinses 1 and 2 and 20 min for rinse 3 at 20 °C). The importance of temperature has also been described by Napper and Thompson [15] in which polyester released more fibers at 40 °C than at 30 °C. In addition, according to a study by Yang et al. [9], when the temperature rises above 60 °C, the amount of microplastics generated increases.

The distribution of the total mass of microplastics during washing and drying is shown in Figure 5. In terms of the fabric-type, the release of microplastics was the highest in acrylic, followed by polyester and nylon. Polyester and nylon generated more microplastics during washing, whereas acrylic generated a larger amount of fibers during drying. It is believed that acrylic generates a larger amount of fibers during drying because many fibers reattach to acrylic during washing. One-way ANOVA was carried out to determine whether differences existed in the amount of microplastics generated by each fabric composition (Table 2). The results indicated that the difference was statistically significant at *F* = 589.33, *p* < 0.001. Therefore, the average amount of microplastics generated by each fabric composition differs.

**Figure 6:**
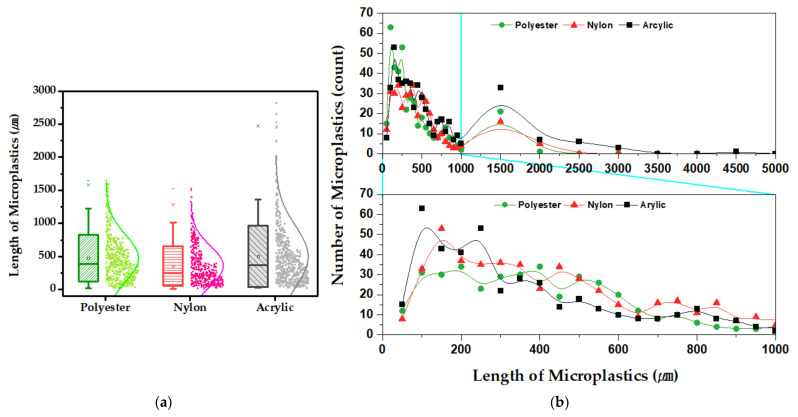
The length and number of microplastic fiber distribution. (**a**) The frequency distribution diagram of microplastic length for each fabric type. (**b**) The number of microplastic fibers of various lengths by fabric type.

Figure 6 shows the fiber length distribution and the number of microplastic fibers released according to the type of fabric. Descriptive statistics for the length of the released microplastic fibers are shown in Table 3. The length of the microplastic fibers released from the polyester, nylon, and acrylic fabrics were 34–1650 μm, 7–1522 μm, and 17–4229 μm, respectively. In other words, the longest fibers were released from the acrylic fabric (4229 μm), whereas the shortest were released from nylon (7 μm). 

For the acrylic fabric, microplastics larger than 1000 μm accounted for 11% of the total microplastic distribution, whereas for nylon and polyester, these long microplastics only accounted for 6% and 4%, respectively. However, relatively short microplastics of less than 500 μm accounted for 59%, 62%, and 71% of the total microplastic distribution for the acrylic, polyester, and nylon fabrics, respectively. For the same external force, the acrylic fabric is easily broken to make relatively large microplastics, whereas nylon is not easily broken, resulting in smaller microplastics. In other words, this result is attributed to the resistance to washing friction being different for each fabric component due to the different physical properties of each fabric.

### 3.2. Physical Properties

The physical properties of fabrics can affect fabric movement during washing and drying owing to the combined result of fabric migration, friction, shape transformation, and restoration. Fuzziness and pilling, flexural stiffness, and tensile strength and strain were measured to derive the physical properties of the fabrics that affected microplastic release.

**Figure 7:**
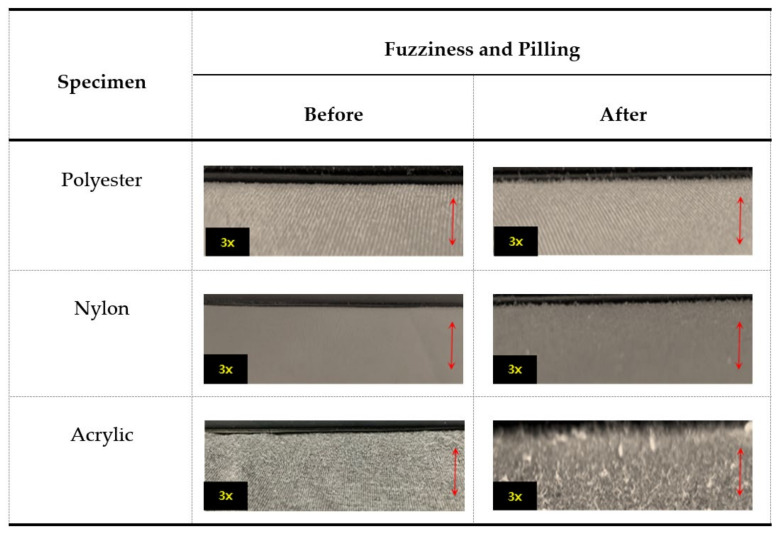
Digital microscopy images (3×) of the fuzziness and pilling on fabric surfaces resulting from washing and drying.

First, because microplastics were generated owing to fabric damage caused by the washing and drying processes, the fuzzing and pilling conditions were observed to compare the damage to fabric surfaces. Fuzzing is a state in which fibers are broken owing to external friction, and fine hairs are formed. Pilling is a state in which the fine hairs or broken fine fibers become entangled and clump together. The surface properties for fuzziness and pilling were graded according to a visual evaluation procedure (Figure 7). The surface observation of polyester and nylon showed purging and some fillings on the fabric surface, which was evaluated as grade 4. In the case of acrylic, purging was observed on the fabric surface, a change in the surface was observed visually, and pills of various sizes were observed over a large area. Accordingly, acrylic was evaluated as grade 2, which was low in terms of visual evaluation. In other words, the amount of microplastics released during the washing and drying processes increased as considerable fuzzing and pilling occurred on the fabric surface. In the case of acrylic, the fabric surface had relatively large areas with fuzzing and pills. Thus, the microplastics generated during the washing process could not escape and were released during the drying process. Hence, the amount of microplastics released during drying was relatively large.

**Figure 8:**
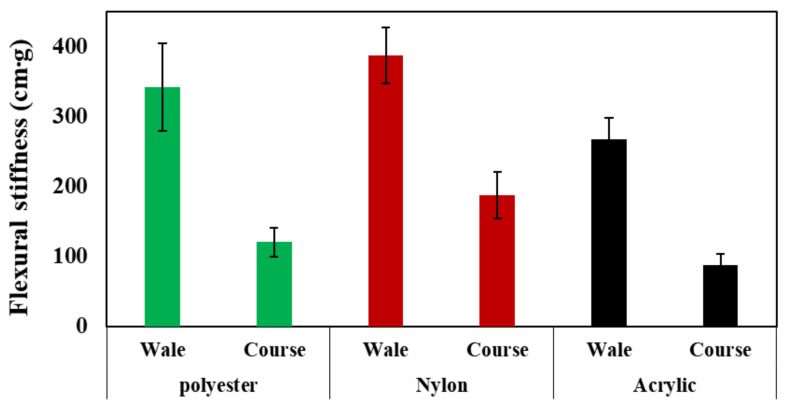
The flexural stiffness according to fabric direction.

The flexural stiffness results of the specimens are shown in Figure 8. Nylon had the highest flexural stiffness, followed by polyester and acrylic, for both the wale and course directions of the fabric. The flexural stiffness of polyester and acrylic was 24% and 42% lower than that of nylon, respectively. Regardless of the fabric type, the flexural stiffness in the course direction was lower than that in the wale direction by 48% in the case of polyester and by approximately 34% in the case of acrylic. Hence, nylon showed the highest bending rigidity and lowest flexibility among the three fabrics. These results were consistent with the trend for the amount of the released microplastics, confirming that the higher the flexural stiffness of the fabric, the lower the amount of microplastics released.

**Figure 9:**
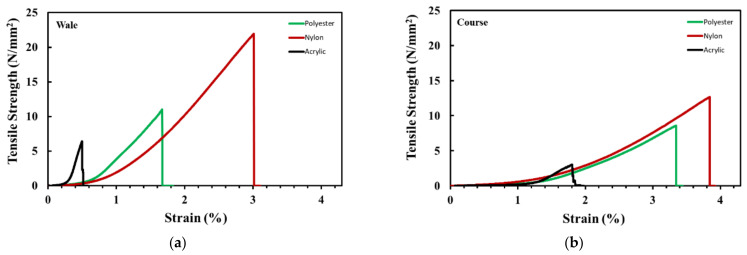
The tensile strength and the strain according to fabric direction. (**a**) Wale. (**b**) Course.

The results for the tensile strength and strain are shown in Figure 9, which were found to be consistent with those for flexural stiffness. For both the wale and course directions, the tensile strength and strain were the highest for nylon, followed by polyester and acrylic. The tensile strength in the wale direction of the fabric was higher than that in the course direction; for acrylic, nylon, and polyester, the difference was 110%, 56%, and 29%, respectively. The tensile strength of polyester and acrylic was 41% and 86% lower than that of nylon, respectively. These results were consistent with the trend for the amount of microplastics released, as also observed in the flexural stiffness results. These results confirmed that the higher the tensile strength of the fabric, the lower the amount of microplastics released. In other words, the amount of the released microplastics was directly affected by the tenacity properties of each synthetic fiber type. The tenacity values for nylon, polyester, and acrylic were 7.5, 7, and 4 g/d, respectively. Pearson’s correlation analysis showed that the correlation between microplastic generation and flexural stiffness was *r* = −0.852 at *p* < 0.01, indicating a negative correlation (Table 4). In addition, the correlation between microplastic generation and tensile strength was *r* = −0.962 and −0.989 at *p* < 0.001, indicating a high negative correlation.

According to the fuzziness and pilling tests, acrylic was evaluated to have the lowest grade, but it generated the largest amount of microplastics. Among the fabrics tested in this study, acrylic consistently shed considerably more fibers than the other two fabric types. This is because acrylic has lower tenacity, and the anchor fibers are easily broken as pills are formed. Nylon has high tenacity; hence, the anchor fibers rarely break to release the pills. Pilling is defined as entangling the fabric surface during wear or washing, forming fiber pills that stand erect on the fabric surface. Fibers from fabrics are lost because of pilling [15,22]. The pill may be worn or pulled away from the fabric because of mechanical action during washing or wear [15,23]. It is important to minimize the pilling tendency to reduce the amount of microplastics released during washing and drying. The rate or extent to which pilling stages occur is determined by the physical properties of the fibers that comprise the fabric [16,24]. Pearson’s correlation analysis showed that the correlation between microplastic generation and fuzziness was *r* = −0.945 at *p* < 0.001, indicating a high negative correlation.

## 4. Conclusions

This study investigated different synthetic fabrics that release fewer microplastics to identify the physical properties of the fabrics that affect fiber release. Accordingly, this study proposed a method to minimize the release of microplastics from fabrics. As washing progressed, all the specimens showed a steady decrease in overall fiber loss. The amount of microplastics released during washing and drying differed according to the polymer type of fabric, which was attributed to differences in the mechanical properties of the fabrics during washing. The amount of microplastics released during washing and drying was the highest for acrylic, followed by polyester and nylon. Polyester and nylon generated more microplastics during washing, whereas acrylic generated a larger amount of fibers during drying. A comparison of the physical properties provided an improved understanding of why different knitted fabrics release different amounts of microfibers. Fabrics with a higher yarn breaking strength, abrasion resistance, and flexural stiffness are expected to have a lower tendency to form fuzz or to release microfibers during the mechanical action of washing.

## Figures and Tables

**Table 1:** Characteristics of the three fabric specimens.

Specimen	Thickness (mm)	Size (m× per 100 g)	Weight (g/m^2^)	Washing Weight (g)	Fiber Diameter (μm)
Polyester	0.55	0.6×0.7	224	500	12.16
Nylon	0.55	0.6×0.7	276		12.70
Acrylic	0.32	1.1×1.6	66		11.74

**Table 2:** Analysis of variance (ANOVA) for factors affecting the release of fibers as a consequence of various fabrics.

	Amount of Microplastics (ppm)					
	*n*	*M*	*SD*	*F*	*p*	Sheffe
Polyester	3	592.07	58.66	589.33	<0.001 ***	n/a **
Nylon	3	198.68	41.18			
Acrylic	3	1428.29	4.98			

** n/a: not applicable, *** *p* < 0.001.

**Table 3:** Descriptive statistics of the fiber length of the released microplastics.

Specimen	Length (µm)						
	*n*	*M*	*SD*	*Md*	*SE*	Min.	Max.
Polyester	488	412.73	306.77	365.87	15.57	34.80	1650.17
Nylon	429	356.73	299.21	248.65	14.45	7.78	1522.81
Acrylic	502	495.75	465.15	371.32	20.76	17.40	4229.52

*n*: number of microplastics, *M*: mean value, *SD*: standard deviation, *Md*: median value, *SE*: standard error, Min: minimum value, Max: maximum value.

**Table 4:** Pearson’s correlation analysis results show correlations between the amount of the released microplastics and physical properties.

	Fuzziness	Stiffness		Tensile Strength	
		Wale	Course	Wale	Course
Total amount of microplastics released (mg/kg)	−0.945 ***	−0.620	−0.852 **	−0.962 ***	−0.989 ***

** p < 0.01, *** *p* < 0.001.

## Data Availability

Data sharing not applicable.
